# Supplementary material for: Common neural and transcriptional correlates of inhibitory control underlie emotion regulation and memory control
Source: Soc Cogn Affect Neurosci. 2020 Jun 8;15(5):523–36. doi: 10.1093/scan/nsaa073 (PMC7328031; doi:10.1093/scan/nsaa073)

Supplementary Material for

Common neural and transcriptional correlates of inhibitory control underlie emotion regulation and memory control

Wei Liu[1], Nancy Peeters[1], Guillén Fernández[1], Nils Kohn[1]

1. Donders Institute for Brain, Cognition and Behaviour, Radboud University Medical Centre, The Netherlands

This PDF file includes:

Supplementary Text

Table S1 to S7

Figure S1 to S4

Correspondence:
Wei Liu
Department of Cognitive Neuroscience
Donders Institute for Brain, Cognition and Behaviour
Radboud University Medical Centre
Trigon Building, Kapittelweg 29
6525 EN Nijmegen, The Netherlands
Tel: +31 (0)24 36 53276
E-mail: W.Liu@donders.ru.nl

1. Comparison between BrainMap and Neurosynth-based co-activation analysis

Co-activation analysis can be performed based on large-scale databases of fMRI studies such as BrainMap or Neurosynth. During the initial data analysis, we took advantages of the relative strengths and weaknesses of the two methods. We augmented the coordinate-based meta-analysis on the Neurosynth with meta-analytic connectivity modelling (MACM) based on the BrainMap to explore co-activation patterns. The idea behind Neurosynth is similar to MACM (which is described in the main text). The Neurosynth algorithm searches for brain regions which co-activate with input coordinates within the same functional contrast, and summarize the results as a co-activation map. Neurosynth has several advantages over BrainMap: (1) Neurosynth is based on automated text mining, therefore includes a higher number of studies and at the same time decreases the potential selection bias of the users. (2) ROI-based MACM is largely dependent on the size (number of voxels) and the shape of the input ROIs. It could be a potential problem because we included far more studies in the ALE analysis of Stop-signal (SS) paradigm and Go/No-go (GN) paradigm compared to the emotion regulation and memory control, leading to on average larger ROIs for stop signal and go/no-go paradigm. On the contrary, Neurosynth-based co-activation analysis does not depend on the ROIs, and it can also base on the MRI coordinates, providing a control for the effect of different size of ROIs. The disadvantage of Neurosynth compared to BrainMap is that automated text mining does not separate different contrasts or experiments within one article. Even though these differences, both methods yielded highly similar co-activation patterns for each ROIs (Figure S1 and Figure S2), suggesting that the effect of methodology and database on the neural network analysis in our study is negligible. We nevertheless chose presented the results of MACM in the main text.

2. Data sharing of non-imaging data via the Open Science Framework (OSF)

We used the OSF database (https://osf.io/6wz2j/) as a venue to share non-imaging data generated within this study.

2.1 Studies and coordinates used in the meta-analyses

For each task (e.g. ER: emotion regulation; TNT: think/no-think; GN: go/no-go; SS: stop-signal), an excel file with all coordinates used in the meta-analyses is uploaded to the folder (ALE_coordinates_data) within the OSF.

2.2 *Code*

Custom python scripts used in this study can be found.

2.3 Details of MACM results

Within the MACM analyses, we estimated in total co-activation patterns of all ROIs from 4 different tasks. Because of the limitation of the number of figures presented in the supplemental materials, we cannot provide all the detailed results for these analyses here. Instead, we used a python package (atlasreader: https://github.com/miykael/atlasreader) to generate coordinate tables and region labels from all co-activation images. All results can be found in an OSF folder (folder name: MACM_results_details, filename: MACM_results_altasreader.zip)

2.4 Lists of genes

Complete gene lists, together with the statistical results, is uploaded in one folder (complete_gene_list) within the OSF database. All files started with “gene_decoding” are the genes whose expression patterns

correlated with the brain activity pattern elicited by each task of interest. All files started with "overlapped_gene_list" are genes whose expression patterns correlated with two (ER and TNT) or more (ER, TNT, GN, and SS) task-related activity patterns simultaneously. Numbers (e.g. 500, 1000) were thresholds used in the analysis to select the genes with most similar spatial patterns. All files started with "difference" are genes whose spatial patterns correlated with inhibition tasks, but not DMN.

2.5 Tables generated by gene ontology enrichment analyses

Tables for enrichment analyses of biological functions (GOEA.zip) or diseases items (disease.zip). Results from different thresholds were presented.

3. Data sharing and 3D visualization of statistical maps via the Neurovault

We uploaded all the statistical maps generated within this study to our Neurovault database (https://neurovault.org/collections/4845/) for data sharing purpose and 3D, interactive visualization. There are in total of 46 maps within the images collection.

- Images names starting with the task names (e.g. ER) and contrast names (e.g. Regulation vs Baseline) are maps resulting from the ALE meta-analysis.
- Images names ending with threshold methods are corrected ALE images (e.g. ALE C05 1K stands for p<.05, uncorrected p<.001, threshold permutations=1000).
- Images names starting with "MACM" are co-activation maps from the MACM analyses.
- No images from the coordinate-based co-activation analysis using Neurosynth were uploaded.

Table S1 Significant activations resulting from the meta-analysis of Go/No-go paradigm

Brain region	Hemisphere	MNI coordinates	Cluster size	Peak voxel Value
Insular/IFG	R	32 16 2	212	0.020847
Inferior temporal gyrus	R	48 -72 -4	98	0.017735
Superior frontal gyrus	R	24 54 10	182	0.018687
Putamen	L	-22 8 6	127	0.019816
DLPFC	R	40 34 24	180	0.021783
IFG, opercular part	R	46 18 32	266	0.024922
Angular	R	50 -54 28	238	0.021796
DLPFC	L	-40 22 38	114	0.021692
Middle cingulate gyrus	R/L	4 10 46	98	0.017317
Inferior Parietal Lobule	R	40 -56 44	200	0.019583
SMA	R/L	0 0 60	95	0.024901

IFG: inferior frontal gyrus; SMA: supplementary motor area; DLPFC: dorsolateral prefrontal cortex

Table S2 Significant activations resulting from the meta-analysis of Stop-signal paradigm

Brain region	Hemisphere	MNI coordinates	Cluster size	Peak voxel Value
Insular/IFG	R	36 20 -4	2050	0.066141
Fusiform	L	-40 -60 -12	252	0.026161
Insular/IFG	L	-38 18 -6	690	0.062398
Inferior parietal lobule	R	48 -44 40	1011	0.038367
Thalamus	R	10 -10 2	812	0.045329
Supramarginal gyrus/ Inferior parietal lobule	L	-58 -46 28	601	0.032832
DLPFC	R	36 46 20	200	0.024995
Middle cingulate gyrus	R/L	2 -24 30	181	0.031727
SMA	R	6 24 34	861	0.042154

IFG: inferior frontal gyrus; SMA: supplementary motor area; DLPFC: dorsolateral prefrontal cortex

Table S3 Estimation of spatial similarity between gene expression maps.

Threshold\Donor	ID9861	ID10021	ID12876	ID14380	ID15496	ID15697
**500**	0.07(0.24)	0.03(0.26)	0.08(0.26)	0.02(0.28)	0.06(0.29)	0.07(0.29)
**1000**	0.07(0.24)	0.03(0.26)	0.09(0.26)	0.03(0.28)	0.07(0.29)	0.08(0.29)
**1500**	0.07(0.24)	0.03(0.26)	0.09(0.26)	0.03(0.28)	0.07(0.29)	0.08(0.29)
**2000**	0.08(0.24)	0.03(0.26)	0.10(0.26)	0.03(0.28)	0.07(0.29)	0.08(0.29)

Mean (standard deviation)

Table S4 Estimation of the significance of overlap genes under the restriction of spatial similarity.

Threshold	Real overlap	Significance	Random overlap (mean)	Random overlap (standard deviation)
**500**	145	P<0.001	12.01	3.32
**1000**	445	P<0.001	48.35	6.62
**1500**	808	P<0.001	108.89	9.7
**2000**	1212	P<0.001	193.28	12.3

Table S5 Estimation of the significance of overlap genes based on permutated statistical maps.

Threshold	Real overlap	Significance	Permutated overlap (mean)	Permutated overlap (standard deviation)
**500**	145	P<0.01	48.62	25.14
**1000**	445	P<0.01	190.41	70.43
**1500**	808	P<0.01	405.04	113.70
**2000**	1212	P<0.01	667.30	147.39

Table S6 Gene Ontology Enrichment Analysis results of the “inhibition-related” genes

GO	NS	enrichment	name	ratio_in_study	ratio_in_pop	p_uncorrected	depth	study_count	p_fdr_bh
GO:0007218	BP	e	neuropeptide signaling pathway	19/779	101/20913	5,3E-09	6	19	3,7E-05
GO:0007268	BP	e	chemical synaptic transmission	30/779	239/20913	6,2E-09	7	30	3,7E-05
GO:0071805	BP	e	potassium ion transmembrane transport	17/779	114/20913	1,1E-06	8	17	0,00434
GO:0007155	BP	e	cell adhesion	38/779	463/20913	5,3E-06	2	38	0,01603
GO:0007165	BP	e	signal transduction	60/779	898/20913	1,2E-05	4	60	0,02976

BP: Biological Processes

Table S7 Disease associations of the "inhibition-related" genes

ID	Name	Source	pValue	FDR B&H	FDR B&Y	Bonferroni	Genes from Input	Genes in Annotation
C0036341	Schizophrenia	DisGeNET Curated	1,92E-07	5,15E-04	4,37E-03	5,15E-04	104	1537
C0005586	Bipolar Disorder	DisGeNET Curated	5,85E-07	7,86E-04	6,66E-03	1,57E-03	61	723
C0001973	Alcoholic Intoxication, Chronic	DisGeNET Curated	4,58E-06	4,10E-03	3,48E-02	1,23E-02	40	396
C0041696	Unipolar Depression	DisGeNET Curated	1,33E-04	8,94E-02	7,58E-01	3,58E-01	39	430
C0014544	Epilepsy	DisGeNET Curated	1,85E-04	8,95E-02	7,59E-01	4,98E-01	47	578
C1269683	Major Depressive Disorder	DisGeNET Curated	2,22E-04	8,95E-02	7,59E-01	5,96E-01	43	509
C0004352	Autistic Disorder	DisGeNET Curated	2,33E-04	8,95E-02	7,59E-01	6,27E-01	48	601
C0751495	Seizures, Focal	DisGeNET Curated	6,19E-04	2,08E-01	1,76E+00	1,66E+00	17	113
C0234533	Generalized seizures	DisGeNET Curated	2,67E-03	7,96E-01	6,75E+00	7,16E+00	16	112
C4317109	Epileptic Seizures	DisGeNET Curated	3,33E-03	8,95E-01	7,58E+00	8,95E+00	15	101
C0004936	Mental disorders	DisGeNET Curated	5,58E-03	1,16E+00	9,84E+00	1,50E+01	29	320
C0525045	Mood Disorders	DisGeNET Curated	8,09E-03	1,16E+00	9,84E+00	2,18E+01	28	309
C0087169	Withdrawal Symptoms	DisGeNET Curated	9,48E-03	1,16E+00	9,84E+00	2,55E+01	12	72
C0027819	Neuroblastoma	DisGeNET Curated	1,15E-02	1,16E+00	9,84E+00	3,09E+01	94	1683
C0422854	Gustatory seizure	DisGeNET Curated	1,26E-02	1,16E+00	9,84E+00	3,38E+01	14	99
C0751123	Atonic Absence Seizures	DisGeNET Curated	1,26E-02	1,16E+00	9,84E+00	3,38E+01	14	99
C0422853	Olfactory seizure	DisGeNET Curated	1,26E-02	1,16E+00	9,84E+00	3,38E+01	14	99
C0270824	Visual seizure	DisGeNET Curated	1,26E-02	1,16E+00	9,84E+00	3,38E+01	14	99

C0751496	Seizures, Sensory	DisGeNET Curated	1,26E-02	1,16E+00	9,84E+00	3,38E+01	14	99
C4505436	Generalized Absence Seizures	DisGeNET Curated	1,26E-02	1,16E+00	9,84E+00	3,38E+01	14	99
C0234535	Seizures, Clonic	DisGeNET Curated	1,26E-02	1,16E+00	9,84E+00	3,38E+01	14	99
C0751110	Single Seizure	DisGeNET Curated	1,26E-02	1,16E+00	9,84E+00	3,38E+01	14	99
C0022333	Jacksonian Seizure	DisGeNET Curated	1,26E-02	1,16E+00	9,84E+00	3,38E+01	14	99
C3495874	Nonepileptic Seizures	DisGeNET Curated	1,26E-02	1,16E+00	9,84E+00	3,38E+01	14	99
C0751056	Non-epileptic convulsion	DisGeNET Curated	1,26E-02	1,16E+00	9,84E+00	3,38E+01	14	99
C0422855	Vertiginous seizure	DisGeNET Curated	1,26E-02	1,16E+00	9,84E+00	3,38E+01	14	99
C4316903	Absence Seizures	DisGeNET Curated	1,26E-02	1,16E+00	9,84E+00	3,38E+01	14	99
C0422850	Seizures, Somatosensory	DisGeNET Curated	1,26E-02	1,16E+00	9,84E+00	3,38E+01	14	99
C0422852	Seizures, Auditory	DisGeNET Curated	1,26E-02	1,16E+00	9,84E+00	3,38E+01	14	99
C1510472	Drug Dependence	DisGeNET Curated	1,30E-02	1,16E+00	9,84E+00	3,49E+01	19	170
C4317123	Myoclonic Seizures	DisGeNET Curated	1,41E-02	1,18E+00	1,00E+01	3,80E+01	14	100
C0270846	Epileptic drop attack	DisGeNET Curated	1,41E-02	1,18E+00	1,00E+01	3,80E+01	14	100
C0011570	Mental Depression	DisGeNET Curated	1,45E-02	1,18E+00	1,00E+01	3,91E+01	41	559
C0086189	Drug Withdrawal Symptoms	DisGeNET Curated	1,70E-02	1,34E+00	1,14E+01	4,57E+01	10	53
C0270844	Tonic Seizures	DisGeNET Curated	1,78E-02	1,36E+00	1,15E+01	4,79E+01	14	102
C0011581	Depressive disorder	DisGeNET Curated	1,86E-02	1,36E+00	1,15E+01	4,99E+01	43	604

Figure S1 Comparisons between BrainMap-based and Neurosynth-based co-activation map for the emotion regulation studies

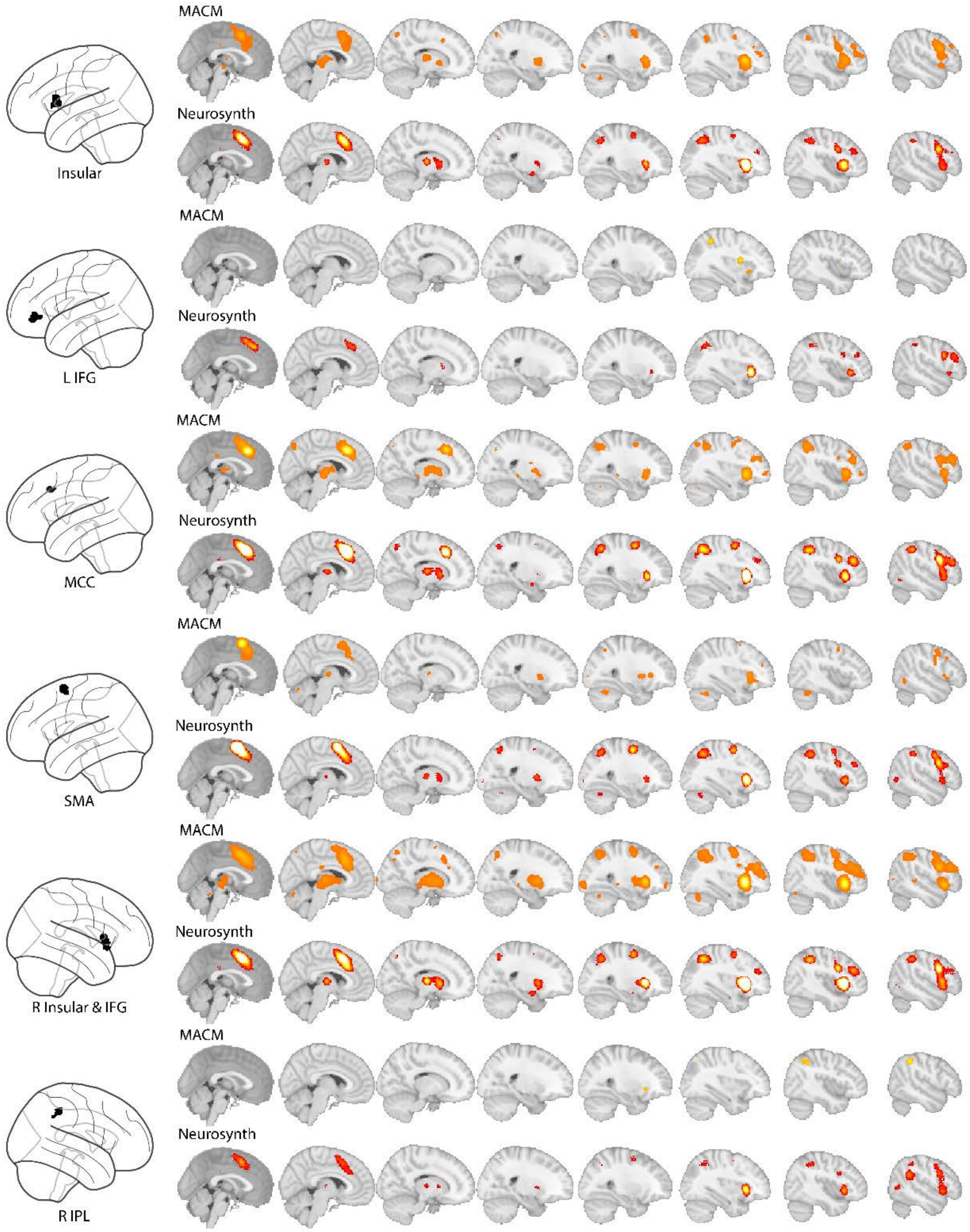

MACM: meta-analytic connectivity modelling; IFG: inferior frontal gyrus; MCC: middle cingulate gyrus; IPL: inferior parietal lobule; SMA: supplementary motor area

Figure S2 Comparisons between BrainMap-based and Neurosynth-based co-activation map for the memory control studies

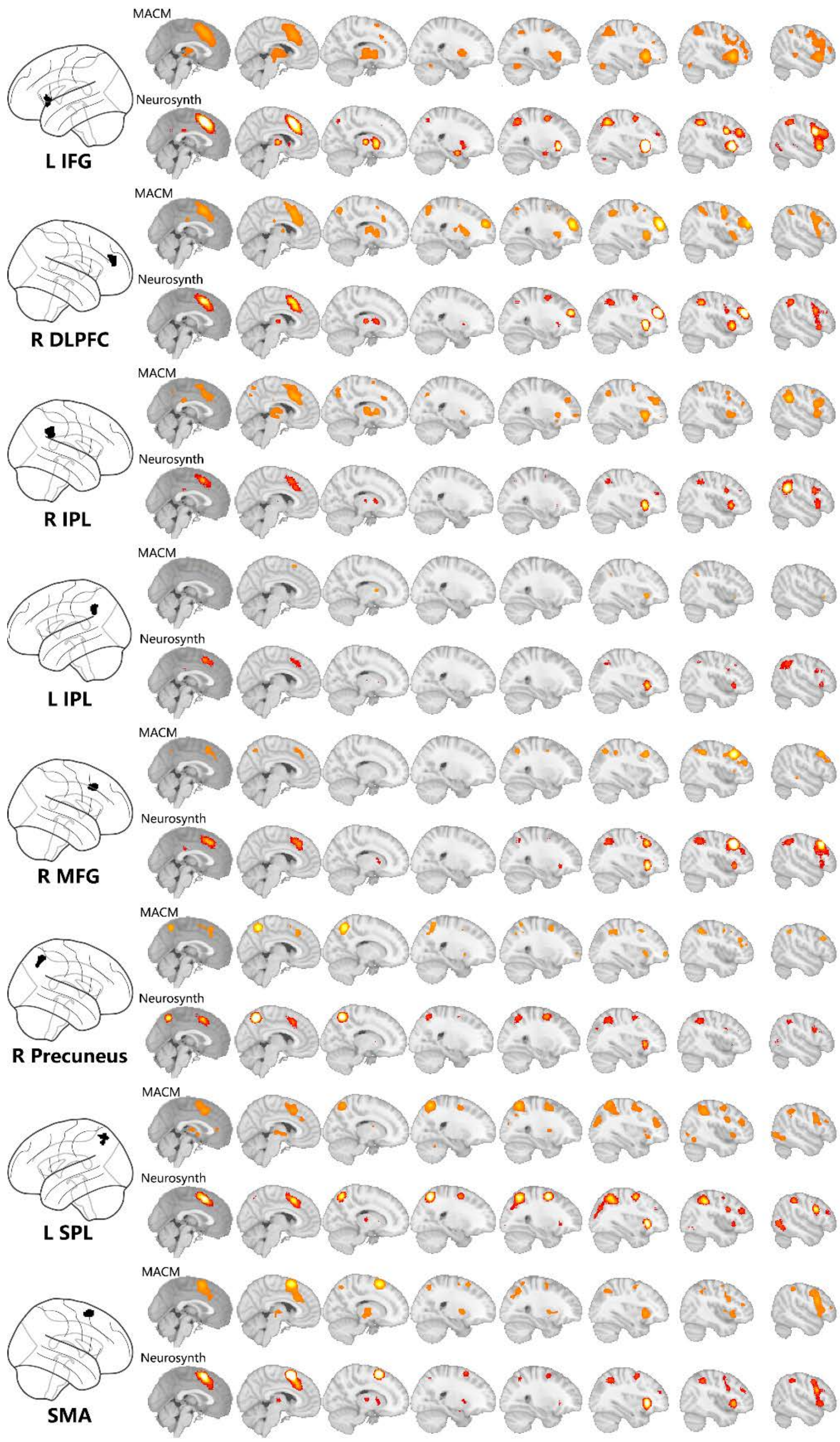

MACM: meta-analytic connectivity modelling; DLPFC: dorsolateral prefrontal cortex; IPL: inferior parietal lobule; SMA: supplementary motor area

Figure S3 ALE meta-analysis of the Default-Mode Network (Task-negative network)

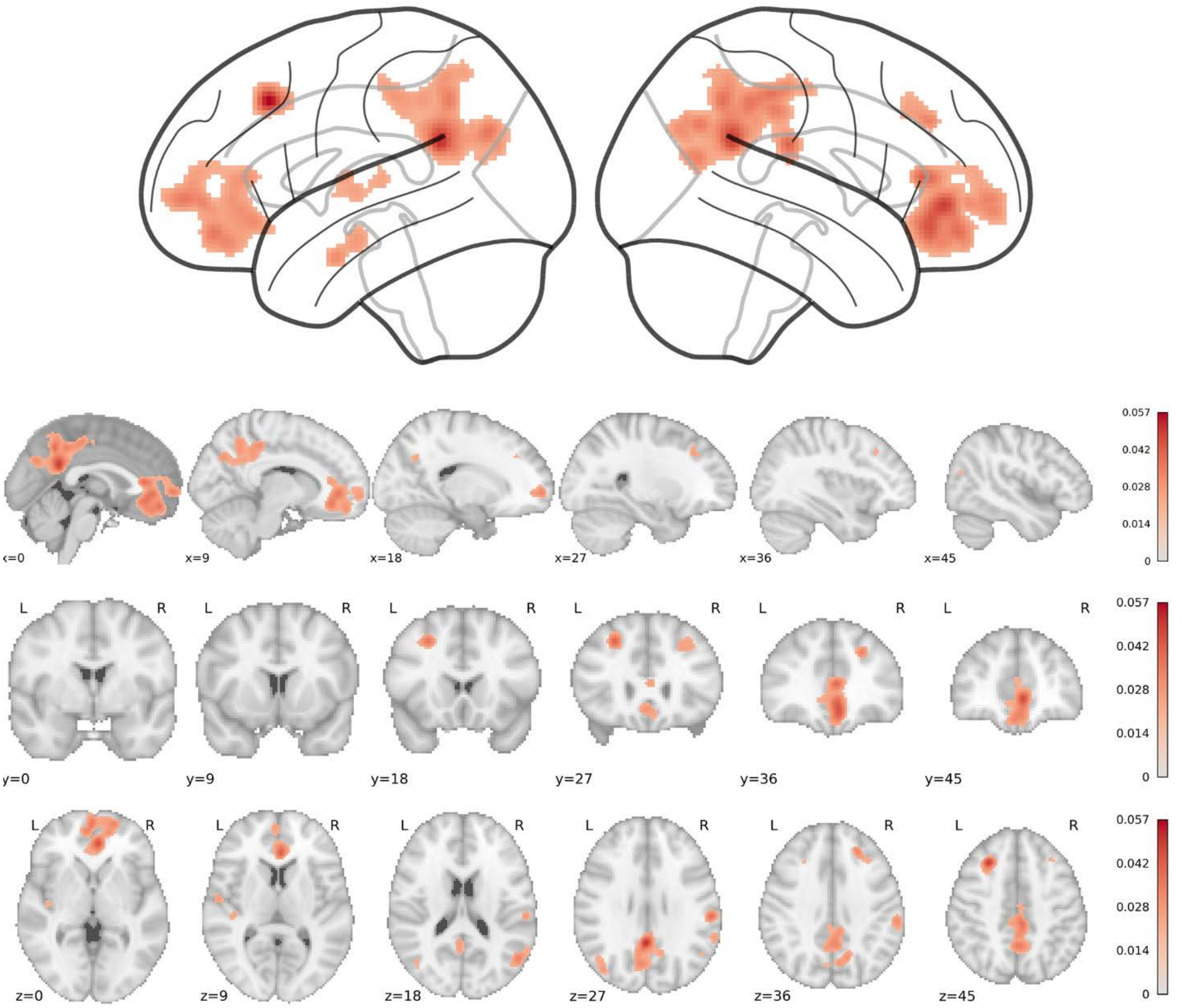

Figure S4 Comparison of diseases association between "inhibition-related" genes and "DMN-related" genes.

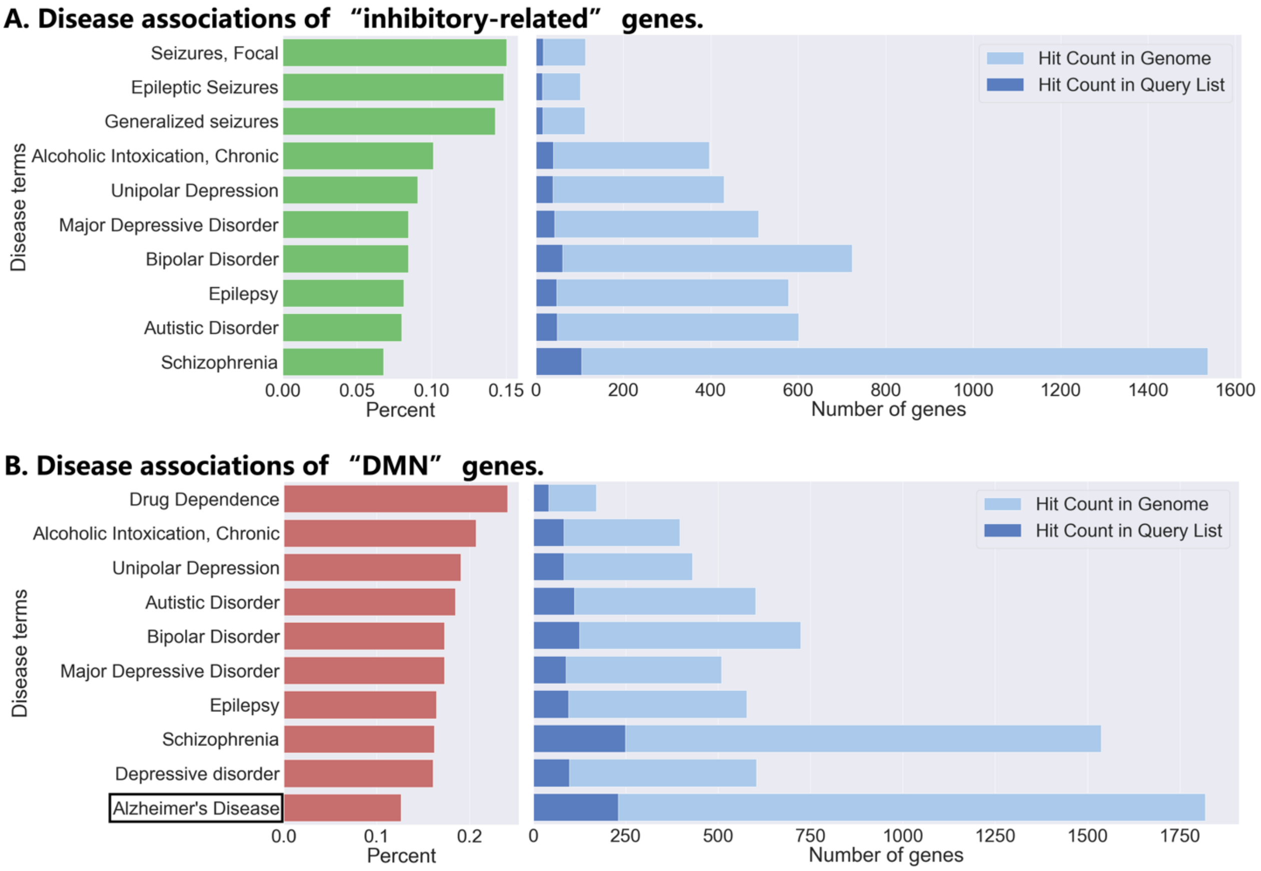

Supplement: scan-20-022-File007_nsaa073 [file scan-20-022-file007_nsaa073.pdf]
